# Impact of the Interdecadal Pacific Oscillation on Tropical Cyclone Activity in the North Atlantic and Eastern North Pacific

**DOI:** 10.1038/srep12358

**Published:** 2015-07-24

**Authors:** Wenhong Li, Laifang Li, Yi Deng

**Affiliations:** 1 Earth and Ocean Sciences, Nicholas School of the Environment, Duke University, Durham, North Carolina, USA; 2 School of Earth and Atmospheric Sciences, Georgia Institute of Technology, Atlanta, Georgia, USA

## Abstract

Tropical cyclones (TCs) are among the most devastating weather systems affecting the United States and Central America (USCA). Here we show that the Interdecadal Pacific Oscillation (IPO) strongly modulates TC activity over the North Atlantic (NA) and eastern North Pacific (eNP). During positive IPO phases, less (more) TCs were observed over NA (eNP), likely due to the presence of stronger (weaker) vertical wind shear and the resulting changes in genesis potential. Furthermore, TCs over NA tend to keep their tracks more eastward and recurve at lower latitudes during positive IPO phases. Such variations are largely determined by changes in steering flow instead of changes in genesis locations. Over the eNP, smaller track variations are observed at different IPO phases with stable, westward movements of TCs prevailing. These findings have substantial implications for understanding decadal to inter-decadal fluctuations in the risk of TC landfalls along USCA coasts.

Tropical cyclones (TCs) are among the most devastating weather systems to affect the United States (US) and Central America. If a TC makes landfall, it can have substantial socio-economic impacts. Thus, understanding and predicting variations and long-term changes in TC movement and frequency is a topic of profound societal significance and intense scientific interest^1^,^2^.

Previous studies have shown that both natural and anthropogenic factors can impact the variability in TC track, although it is premature to conclude that human activities have caused detectable changes^3^. Using fine-resolution global atmospheric models, Murakami and Wang (2010)^4^ suggested that warmer climate due to anthropogenic forcing is projected to reduce probabilities of TC landfall over the Southeastern U.S., but increase the influence of TCs on the Northeastern U.S., mainly due to changes in TC genesis locations. North-Atlantic natural modes of climate variability could also modulate TC tracks over the basin. For example, a negative North Atlantic Oscillation (NAO) tends to be associated with more TCs making landfall along the US East Coast, especially those TCs that form outside of the main hurricane development region (10°–25°N, 80°–20°W)^5^,^6^,^7^,^8^,^9^,^10^,^11^. The Atlantic Meridional Mode (AMM) also has a significant correlation with North Atlantic TC activity, particularly its frequency^12^,^13^. By inducing the AMM at decadal scales, the Atlantic Multi-decadal Oscillation (AMO) also influence TC activity. Additionally, Pacific modes, such as the El Nino Southern Oscillation (ENSO), impact TC activity over the North Atlantic and eastern Pacific^14^,^15^,^16^,^17^,^18^,^19^,^20^,^21^. La Niña appears to increase hurricane landfall relative to neutral years from Georgia northward in the U.S.^16^, and more hurricanes make landfall in Florida than along the East coast during neutral years^14^.

However, whether and how Pacific decadal modes, specifically the Interdecadal Pacific Oscillation (IPO)^22^ (See *Methods* for more detail) influences the variabilities of TC frequency and track in the North Atlantic and eastern North Pacific has not been *systematically* investigated although a few studies discussed multi-decadal variability of vertical wind shear over tropical Atlantic and central Pacific^23^,^24^. The current work aims to fill this gap in the knowledge base. Results presented here will help to untangle the interplay between long-term changes in the TC activity due to the natural forcing and anthropogenic climate change. This will eventually lead to improved TC predictions and projections over the North Atlantic and eastern North Pacific, especially in the near-term predictions.

## Results

This study mainly addresses the variations of TC activity over the North Atlantic and eastern North Pacific, respectively, during different IPO phases using the best-track data compiled by the National Hurricane Center^25^ for the period 1949–2012 when TC data are available over both basins (See *Methods* for details). The analysis considers TCs with tropical storm intensity (i.e., Maximum sustained winds greater than 39 mph) and stronger. Corresponding large-scale environmental flows are analyzed using the NCEP/NCAR reanalysis.

### IPO impact on TC frequency

Table 1 shows variations in TC frequency at different IPO phases over the two basins. On average, there are 7.3 TCs (4.5 hurricanes) per year over the North Atlantic when IPO index is negative. TC frequency decreases to 4.3 per year during IPO positive phases, about 70% reduction compared to those in IPO negative phases. The correlation coefficient between the North Atlantic TC frequency and the IPO index is −0.27, significant at the 95% level.

Compared to the North Atlantic, variations of TC frequency over the eastern North Pacific are the opposite during different IPO phases. On average, 18.8 TCs (10.3 hurricanes) per year can be observed during the IPO positive phase. However, during the IPO negative phases, only 8.8 TCs (4.7 hurricanes) per year were found over the region, an 82% drop of TC numbers compared to the IPO positive phase. The correlation between the eastern North Pacific TC frequency and the IPO index is 0.42, significant at the 99% level.

### IPO impact on TC track variabilities

#### a) North Atlantic

**Figure 1:**
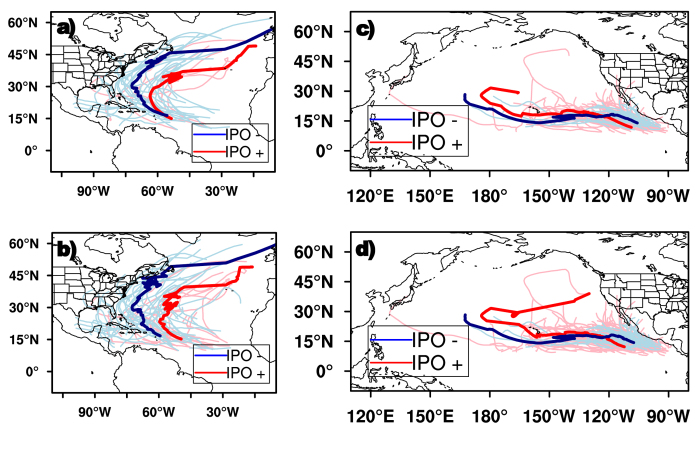
IPO impacts on TC and hurricane Tracks. Track composite of hurricane and all TCs (tropical storms and hurricanes), respectively, during IPO positive (red) and negative (blue) phases over the North Atlantic (**a,b**) and eastern North Pacific (**c,d**). The maps in Fig. 1 were generated using NCAR Command Language (NCL) version 6.3.0, open source software free to public, by UCAR/NCAR/CISL/TDD, http://dx.doi.org/10.5065/D6WD3XH5.

Figure 1 shows the track composite of hurricanes and all TCs (tropical storms and hurricanes), respectively, at different IPO phases (see *Methods* for details). TCs become hurricanes at similar locations during both positive and negative IPO phases, around 50^o^W, 15^o^N (Fig. 1a). During the negative IPO phase, hurricanes tend to move west and northwestward from their first identified region (50^o^W, 15^o^N), then recurve at around 73^o^W, 30^o^N and finally move north to northeastward in the subtropics and mid-latitudes (Fig. 1a). Similarly, hurricanes first move west and northwestward in the tropics during positive IPO phases; but the hurricane tracks stay 5–10 degrees east compared to those during negative IPO phases, and the hurricanes tend to recurve at lower latitudes, about 20^o^ to 25^o^N (Fig. 1a).

Similar changes in TC movement can be observed when comparing TCs in general (all tropical storms plus category 1–5 hurricanes) at different IPO phases (Fig. 1b). Specifically, during IPO positive phases, TCs tend to move west/northwestward from the genesis region, then recurve north/northeastward east of 60^o^W (Fig. 1b). TC tracks during the negative IPO phases are akin to those observed in positive IPO phases except that the composite track is about 10–15 degrees westward. We also notice that TCs are generated at slightly different regions at different IPO phases (about 6 degrees apart from the composite tracks) over the North Atlantic (Fig. 1b), but the difference is not significant at the 99% level.

#### b) Eastern North Pacific

Over the eastern North Pacific, TCs form in similar locations (15^o^N, 110^o^W) at different phases of IPO. Unlike those over the North Atlantic (Fig. 1a,b), tracks of TCs (hurricanes) over the eastern North Pacific are similar during positive and negative IPO phases, except that TC (hurricane) motions are stably confined in the tropics and their tracks extend further westward during IPO negative phase (Fig. 1c,d).

What might cause the changes of TC activity over the North Atlantic and eastern North Pacific during different IPO phases? In order to understand the factors/processes contributing to the changes in TC track and frequency from one phase of the IPO to the other, we analyzed the main environmental factors responsible for variations of TC activity, including steering flows^26^, genesis potential index (GPI)^27^,^28^, and vertical wind shear^28^ in the section.

#### a) Factors responsible for the variability in TC frequency

**Figure 2:**
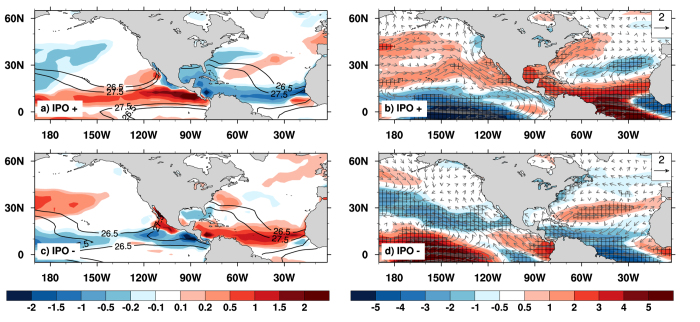
IPO influence on large-scale environmental factors for TC and Hurricane frequency. (**a,b**) GPI (shaded) and SST (contour, unit: ^o^C); (**c,d**) magnitude of wind shear anomalies (shaded) and the shear anomalies (vector, unit: m s^−1^) during the IPO positive (top) and negative (bottom) phases. Stippling indicates regions exceeding 90% statistical confidence. Figure 2 is generated using NCAR Command Language (NCL) version 6.3.0, open source software free to public, by UCAR/NCAR/CISL/TDD, http://dx.doi.org/10.5065/D6WD3XH5.

Figure 2 illustrates variations of GPI and vertical wind shear anomalies at different IPO phases over the two basins. GPI is an empirical index that is developed to assess TC frequency from large-scale analyses^27^,^28^, and can reasonably capture observed seasonal variations and the location of TC genesis^15^,^29^,^30^,^31^. During the positive phase of IPO, GPI tends to decrease over the tropical North Atlantic, partly accounting for the decreased frequency of TC in the region (Fig. 2a). The decreased GPI is mainly caused by an enhanced vertical wind shear (Fig. 2b) since sea surface temperatures (SSTs) do not change substantially over the tropical North Atlantic across different IPO phases (Fig. 2a,c). Over the eastern North Pacific, the opposite situation occurs where decreased vertical wind shear leads to increased GPI (Fig. 2a) that favors more frequent TC formation (Table 1).

During the negative IPO phases, the eastern North Pacific has fewer TC formations due to decreased GPI (Fig. 2c), which results from both an increased wind shear (Fig. 2d) and decreased SST over the region (Fig. 2c). Over the North Atlantic, on the contrary, decreased wind shear (Fig. 2d) leads to an increased GPI (Fig. 2c), in favor of more TC formation. These changes occurring at decadal timescales resemble interannual fluctuations in TC activity found over the North Atlantic during ENSO years, i.e., TC formation is suppressed in El Nino years^4^,^6^,^9^,^16^.

#### b) Processes responsible for variability in TC tracks

**Figure 3:**
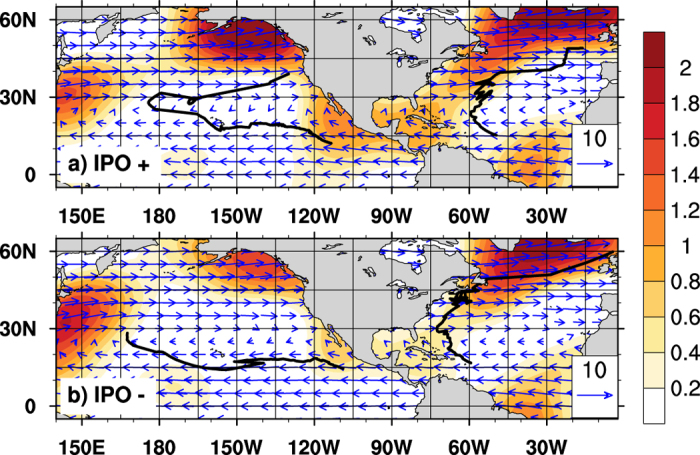
IPO influence on basin-scale steering flows. Large-scale steering flows (vectors, unit: m s^−1^), magnitude of meridional wind (shaded, unit: m s^−1^), and track composite of TCs over North Atlantic and eastern North Pacific during the IPO positive (top) and negative (bottom) phases. Horizontal smoothing is applied to steering flow so that the spherical harmonic components with wavenumber larger than 11 is filtered out. Figure 3 is generated using NCAR Command Language (NCL) version 6.3.0, open source software free to public, by UCAR/NCAR/CISL/TDD, http://dx.doi.org/10.5065/D6WD3XH5.

Figure 3 shows the composite of TC steering flows over the two basins at different IPO phases. During the positive IPO phase, large-scale, anticyclonic steering flows are mainly easterlies in the tropical North Atlantic (Fig. 3a), causing TCs to move westward. Strong southerly winds can be observed along 60^o^W, driving the northward movement of TCs around 60^o^W, 20^o^N (Fig. 1). TCs recurve between 20^o^N and 25^o^N and then move eastward north of 25^o^N. On the contrary, during negative IPO phases, large-scale anticyclonic steering flow is located more to the west and slightly to the north (Fig. 3b) compared to that during IPO positive phases (Fig. 3a). Southerly winds are only observed westward of 70^o^W. The large-scale environmental flows thus steer TCs further westward, and cause them to recurve at higher latitudes (25–30^o^N) during negative IPO phases (Fig. 3b).

Over the eastern North Pacific, there is no significant difference in large-scale steering flows at different IPO phases (Fig. 3a,b) except that much stronger tropical easterlies are observed during IPO negative phases. The anomalously strong easterlies likely reflect strengthened anticyclonic circulations in the region (Fig. 3b), responsible for a stably confined, more westward motion of TCs during the period.

Murakami and Wang (2010)^4^ found that model-projected changes in TC tracks over the North Atlantic are not due to changes in large-scale steering flows, but rather due to changes in TC genesis location in a warming climate. We thus compared the locations of TC genesis during different IPO phases. Over the eastern North Pacific, TCs formed at similar regions (Fig. 1c,d), around 15^o^N, 105–110^o^W during different IPO phases, thus the TC track difference is mainly a result of changing steering flows in the region. Over the North Atlantic, hurricanes are also identified at similar locations during positive and negative IPO phases (Fig. 1a), but tropical storms usually form 6 degrees apart (Fig. 1b), although the difference of the genesis locations is not significant. This indicates that the Pacific decadal mode may influence TC tracks mainly through altering large-scale steering flow. The conclusion can also be validated by comparing recurvature latitudes of the composite TC tracks over the North Atlantic during positive and negative IPO phases. The steering flow changes from southerly between 20°–25°N to westerly north of 25°N in the IPO positive phase (Fig. 3a), and most TCs recurve around 20°–25°N. During the IPO negative phase, dominant winds change from easterly to southerly at 70°W, 25°–30°N, and then to westerly north of 30°–35°N (Fig. 3b), corresponding to a northward shift of TC recurving areas (Fig. 1b).

## Discussion

**Figure 4:**
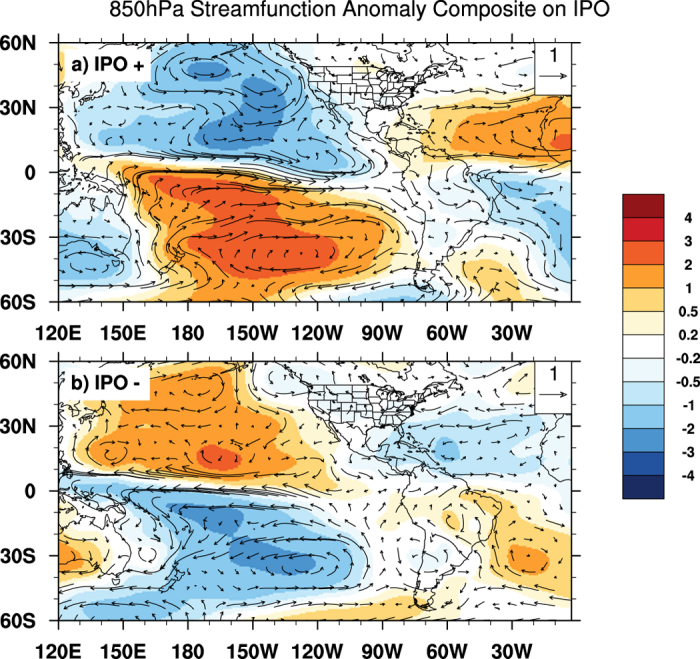
Large-scale atmospheric circulation in response to IPO. 850 hPa stream function anomaly (shaded; unit: m^2^ s^−1^) and horizontal wind anomaly (vector; unit: m s^−1^) during the IPO positive (top) and negative (bottom) phases. Horizontal smoothing is applied to both stream function and horizontal wind so that the spherical harmonic components with wavenumber larger than 11 is filtered out. Figure 4 is generated using NCAR Command Language (NCL) version 6.3.0, open source software free to public, by UCAR/NCAR/CISL/TDD, http://dx.doi.org/10.5065/D6WD3XH5.

Why can the Pacific decadal mode influence wind and thus TC activity over North Atlantic and eastern North Pacific? Fig. 4 shows the composite 850 hPa stream function anomaly during different IPO phases. Corresponding to the Pacific heating associated with positive IPO, a typical Gill–Matsuno-type response^32^,^33^ of the atmosphere can be observed over the tropical Atlantic and Pacific (See Supplementary for the middle and upper level circulation). Equatorial Kelvin waves lead to circulation anomalies to the east of the heating whereas equatorial Rossby waves are responsible for the circulation changes occurring west of the heating center. At 850 hPa, intensified easterlies to the east of the heating are a prominent feature of such a response and are observed over the tropical Atlantic during positive IPO phases (Fig. 4a). The anticyclonic circulation anomalies over the eastern tropical and subtropical Atlantic are likely related to the large-scale diabatic cooling over North Africa induced by Sahel droughts, which have been shown to occur more often during positive IPO phases^34^. The circulation during the negative IPO phase is generally opposite to that in the IPO positive phase except that the Atlantic signal is slightly weaker (Fig. 4b). These results indicate that the Pacific decadal mode modulates TC activity over both basins by changing large-scale circulations in the regions.

## Conclusions

Tropical cyclone frequency and movements over the North Atlantic and eastern North Pacific are of great socioeconomic significance to Central and North America. Previous studies investigated the impacts of the Atlantic natural modes and ENSO on TC activity over the regions. The results presented here demonstrate that the IPO also exerts profound impacts on the track and frequency of TCs over both the North Atlantic and eastern North Pacific basins. Specifically, positive phases of the IPO correspond to a significant decrease (increase) of TC numbers over the North Atlantic (eastern North Pacific) that is mainly driven by changes in wind shear and GPI. The IPO also influences TC movements: during the positive phase, TCs over the North Atlantic tend to keep their tracks eastward and recurve at lower latitudes compared to those during the negative IPO phases; over the eastern North Pacific, IPO leads to relatively small changes in the TC tracks. It is further shown that changes in the composite tracks at different IPO phases are largely determined by differences in steering flow patterns instead of genesis locations. The large-scale circulation changes that give rise to different steering flow patterns over both basins are in turn consistent with Gill-Matsuno-type responses excited by anomalous, SST-related diabatic heating in the Pacific and by the anomalous latent heating over the North Africa (e.g., those associated with the Saharan drought in IPO positive phases).

## Methods

The hurricane track data is obtained from the best track data (HURDAT2)^25^, which are available from http://www.nhc.noaa.gov/data/#hurdat. Previous studies showed that total hurricane counts for the North Atlantic became fairly reliable after aircraft reconnaissance began in 1944^35^; over the eastern North Pacific, HURDAT2 from the National Hurricane Center are *only* available after 1949^36^. Atmospheric circulation regimes are derived from NCEP/NCAR reanalysis datasets^37^. The variables analyzed in this study include tropospheric wind, air temperature, relative humidity, and surface pressure. We thus choose the period 1949–2012 to analyze the large-scale environment fields for TCs over both basins.

NOAA Extended Reconstructed SSTs are used to calculate the IPO index. The IPO is a multidecadal scale SST pattern similar to ENSO, but is more symmetric about the equator and has more loadings in both North and South Pacific^22^. An empirical orthogonal function (EOF) analysis is applied to global SST to extract the IPO mode. Prior to EOF analysis, Lanczos filter (cut-off frequency is set to 0.3 years^-1^) is used to remove the high-frequency SST variability and thus obtain the decadal to multi-decadal scale variation of SST, following Dai (2013)^38^.

**Figure 5:**
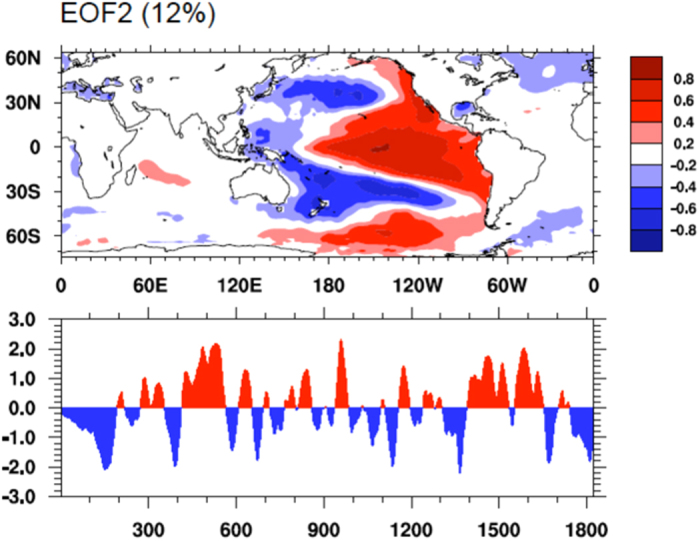
IPO index construction. The second leading empirical orthogonal functions (EOF) of the 3-year moving averaged SSTs from the NOAA Extended Reconstructed SST data set as in Dai (2013). Bottom panel shows the time series of the IPO index derived by applying the 9-year moving averaging twice to the (3-year smoothed) annual series. The percentage variance explained by the EOF is shown on top of panel. Figure 5 is generated using NCAR Command Language (NCL) version 6.3.0, open source software free to public, by UCAR/NCAR/CISL/TDD, http://dx.doi.org/10.5065/D6WD3XH5.

EOF analysis indicates that the first global SST mode reflects a planetary-scale warming (not shown). The second mode shows a typical IPO pattern with ENSO-like SSTA pattern in the tropical Pacific and substantial loading over the extra-tropical oceans (Fig. 5). The EOF mode 2 obtained in this study highly resembles those of the IPO modes as defined in various studies, such as Power *et al.* (1999)^22^ and Dai (2013)^38^.

To study the impact of IPO on TC genesis and movement, steering flow, vertical wind shear, and genesis potential index (GPI) are calculated. The steering flow is defined as the mass-weighted average of horizontal wind between 850 hPa and 500 hPa^26^, while the wind shear is the wind difference between 850 hPa and 200 hPa^28^. GPI is originally developed by Emanuel and Nolan (2004)^28^, which is formulated as:


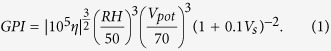


In Eq. (1), 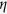 is 850 hPa absolute vorticity (s^−1^); RH is relative humidity (%) at 700 hPa; and 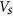 is the magnitude of vertical wind shear between 850 hPa and 200 hPa. 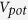 is maximum potential intensity as defined in Emanuel (1995)^27^:


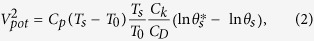


where 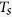 is SST (K) and 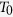 is air temperature at tropopause (K). 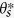 and 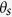 is surface saturation equivalent potential temperature and equivalent potential temperature, respectively. Equations (1) and (2) suggest that GPI is sensitive to the changes in SST and the vertical wind shear; we thus analyze the contributions of IPO induced SST and wind shear to GPI over the two ocean basins. Specifically, the contribution of wind shear to GPI is quantified by setting SST to JJASON climatology in Eq. (2). In contrast, the contribution of SST is quantified by setting 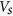 to 1949–2012 climatology in Eq. (1).

Our analysis suggests that the influence of IPO on GPI and thus TC frequency is mainly through the changes in wind shear. Over the eastern North Pacific, changes in wind shear contribute to 70% of the GPI changes, while the SSTA contributes the remaining 30%. In addition, the wind shear contributes 108% GPI change over the North Atlantic basin, and thus the contribution of SSTA is negligible.

We use the composite method to highlight the features of TC activity during IPO positive and negative phases, separately. To ensure the same number of composite samples in both cases, we select TC tracks (and all other key variables such as GPI, wind shear, and steering flow) using the following method. For the 64 (1949–2012) hurricane seasons (June to November, JJASON), TC tracks are first arranged according to IPO index values from positive (i.e., IPO positive phases) to negative (IPO negative phases). All variables related to TC activity (such as TC tracks, GPI etc) corresponding to the top (bottom) 10-percentile of the JJASON IPO index are averaged to highlight the common features of TC activity during IPO positive (negative) phases. The composite results are not sensitive to difference percentile criteria (such as 15%, 20%, not shown).

## Additional Information

**How to cite this article**: Li, W. *et al.* Impact of the Interdecadal Pacific Oscillation on Tropical Cyclone Activity in the North Atlantic and Eastern North Pacific. *Sci. Rep.* **5**, 12358; doi: 10.1038/srep12358 (2015).

## Supplementary Material

Supplementary Information

## Figures and Tables

**Table 1:** Variations of TC (hurricane) number per year during different IPO phase.

	IPO +	IPO −
Hurricanes over E. North Pacific	10.3	4.7
Hurricanes over North Atlantic	1.3	4.5
TCs over E. North Pacific	18.8	8.8
TCs over North Atlantic	4.3	7.3
