# Supplementary material for: Impact of the Interdecadal Pacific Oscillation on Tropical Cyclone Activity in the North Atlantic and Eastern North Pacific
Source: Sci Rep. 2015 Jul 24;5:12358. doi: 10.1038/srep12358 (PMC4648449; doi:10.1038/srep12358)
Supplement: Supplementary Information [file srep12358-s1.doc]

**Impact of the Interdecadal Pacific Oscillation on Tropical Cyclone Activity in the North Atlantic and Eastern North Pacific**

Wenhong Li, Laifang Li, and Yi Deng

**Figure S1**. Same as Fig. 4 in the main text but for 500hPa. The figure is generated using NCAR Command Language (NCL) version 6.3.0, open source software free to public, by UCAR/NCAR/CISL/TDD, <http://dx.doi.org/10.5065/D6WD3XH5>

**Figure S2.** Same as Fig. 4 in the main text but for 200hPa. The figure is generated using NCAR Command Language (NCL) version 6.3.0, open source software free to public, by UCAR/NCAR/CISL/TDD, <http://dx.doi.org/10.5065/D6WD3XH5>
